# P03 Comparative evaluation of antifungal susceptibility testing by a commercial system with broth microdilution method for *Candida auris*

**DOI:** 10.1093/jacamr/dlag102.009

**Published:** 2026-06-26

**Authors:** Bharat Chandra Das, Md Nizam Ahmed, Madhavi Kirti, Aarsi, Parul Singh, Purva Mathur

**Affiliations:** Microbiology Unit, Jai Prakash Narayan Apex Trauma Centre, AIIMS, New Delhi; Microbiology Unit, Jai Prakash Narayan Apex Trauma Centre, AIIMS, New Delhi; Microbiology Unit, Jai Prakash Narayan Apex Trauma Centre, AIIMS, New Delhi; Microbiology Unit, Jai Prakash Narayan Apex Trauma Centre, AIIMS, New Delhi; Microbiology Unit, Jai Prakash Narayan Apex Trauma Centre, AIIMS, New Delhi; Microbiology Unit, Jai Prakash Narayan Apex Trauma Centre, AIIMS, New Delhi

## Abstract

**Background:**

*Candida auris* is an MDR yeast causing invasive candidiasis with high mortality (29%–62%). It also has an outbreak potential in healthcare settings. Accurate antifungal susceptibility testing (AFST) is critical due to its very high resistance profile to azoles, polyenes, and echinocandins. The absence of *C. auris* specific breakpoints, complicates patient’s treatment. Commercial automated antimicrobial susceptibility testing (AST) platforms do not show reliable results. So, the need for a feasible, reliable, technically non-demanding process for AFST is need of the hour.

**Objective:**

This study is to evaluate a commercial system (Sensititre YeastOne from Thermo Scientific) with gold standard broth microdilution (BMD) method.

**Methods:**

This study was conducted at a Level-1 trauma centre in India from 2024 to 2025. It is a tertiary care facility and manages a high volume of critically ill patients, including those with severe trauma, surgical complications, and nosocomial infections, making it a relevant setting for studying *C. auris* outbreaks. Samples were collected from patients in intensive care units (ICUs) and surgical wards. Primarily blood, urine, and wound samples were collected, reflecting common sources of invasive candidiasis. Twenty *C. auris* isolates were obtained from clinical samples and were identified using VITEK-MS (MALDI-TOF MS) system. This method was chosen as phenotypic characteristics cannot reliably distinguish *C. auris* from other *Candida* species. AFST was done using two phenotypic methods: Sensititre-system and gold standard BMD. Sensititre-system is a US Food and Drug Administration (US-FDA) approved automated BMD method for AFST. It utilizes 96-well microtitre plates. Wells are precoated with increasing concentration of different antifungals and resazurin as indicator of growth. It is used for in vitro diagnostic (IVD) and research use only (RUO) purposes. All isolates were cultured on Sabouraud Dextrose Agar (SDA) at 37°C for 24 h prior to testing. *Candida parapsilosis* (ATCC: 22019) and *Candida krusei* (ATCC: 6258) were used as quality control strains. 0.5 McFarland turbidity of inoculum was prepared by using densitometer. US centres for disease control and prevention (CDC) tentative breakpoints applied (e.g. fluconazole ≥32 mg/L, amphotericin B ≥2 mg/L, anidulafungin ≥4 mg/L, caspofungin ≥2 mg/L, micafungin ≥4 mg/L). Sensitivity, specificity, positive predictive value (PPV), negative predictive value (NPV) was calculated.

**Results:**

Fluconazole susceptibility: Sensititre system showed 100% concordance with gold standard BMD test. All isolates were fluconazole-resistant by both methods. Caspofungin, micafungin and anidulafungin susceptibility: There was 100% concordance between these two tests. All isolates were anidulafungin susceptible by both methods. Amphotericin B susceptibility: 2 out of 20 samples were resistant by both methods. There was one false positive and one false negative result by Sensititre-system. Cohen’s kappa value showed moderate agreement of gold standard method with proposed method (κ=0.44) with sensitivity of 94%, specificity of 50%, PPV of 94% and 50% NPV.

**Conclusions:**

The Sensititre system demonstrated, susceptibility results of fluconazole and echinocandins with high accuracy. But in comparison with gold standard BMD method, amphotericin B susceptibility results showed moderate agreement. These findings highlight the need for standardized *C. auris* specific breakpoints and improved AFST methods to guide therapy in outbreak-prone settings.

